# The regulation of TRPA1 expression and function by Th1 and Th2-type inflammation in human A549 lung epithelial cells

**DOI:** 10.1007/s00011-023-01750-y

**Published:** 2023-06-29

**Authors:** Samu Luostarinen, Mari Hämäläinen, Antti Pemmari, Eeva Moilanen

**Affiliations:** The Immunopharmacology Research Group, Faculty of Medicine and Health Technology, Tampere University and Tampere University Hospital, Tampere, Finland

**Keywords:** TRPA1 cation channel, Inflammation, Th1-Th2 balance, Janus kinase inhibitors, STAT6 transcription factor, Glucocorticoids

## Abstract

**Background:**

Transient Receptor Potential Ankyrin 1 (TRPA1) is a cation channel that mediates pain, itch, cough, and neurogenic inflammation in response to pungent compounds such as acrolein in cigarette smoke. TRPA1 is also activated by endogenous factors and promotes inflammation in asthma models. We have recently shown that TRPA1 is upregulated by inflammatory cytokines in A549 human lung epithelial cells. Here, we explored the effects of Th1 and Th2-type inflammation on TRPA1.

**Methods and results:**

TRPA1 expression and function was studied in A549 human lung epithelial cells. To induce inflammation, the cells were exposed to a combination of cytokines TNF-α and IL-1β; and to model Th1 or Th2-type responses, IFN-γ or IL-4/IL-13 was added, respectively. TRPA1 expression (measured by RT-PCR and Western blot) and function (assessed by Fluo-3AM intracellular calcium measurement) was enhanced under the influence of TNF-α + IL-1β. IFN-γ further enhanced TRPA1 expression and function, whereas IL-4 and IL-13 suppressed them. The effects of IFN-γ and IL-4 on TRPA1 expression were reversed by the Janus kinase (JAK) inhibitors baricitinib and tofacitinib, and those of IL-4 also by the STAT6 inhibitor AS1517499. The glucocorticoid dexamethasone downregulated TRPA1 expression, whereas the PDE4 inhibitor rolipram had no effect. Under all conditions, TRPA1 blockade was found to reduce the production of LCN2 and CXCL6.

**Conclusions:**

TRPA1 expression and function in lung epithelial cells was upregulated under inflammatory conditions. IFN-γ further increased TRPA1 expression while IL-4 and IL-13 suppressed that in a JAK-STAT6 dependent manner which is novel. TRPA1 also modulated the expression of genes relevant to innate immunity and lung disease. We propose that the paradigm of Th1 and Th2 inflammation is a major determinant of TRPA1 expression and function, which should be considered when targeting TRPA1 for pharmacotherapy in inflammatory (lung) disease.

**Supplementary Information:**

The online version contains supplementary material available at 10.1007/s00011-023-01750-y.

## Introduction

Transient Receptor Potential Ankyrin 1 (TRPA1) is a neuronal cation channel activated by noxious compounds [1, 2]. TRPA1 permeates Na^+^, Ca^2+^ and other cations and mediates pain, itch, cough, and neurogenic inflammation [1, 2]. Exogenous TRPA1 activators include, for example, allyl isothiocyanate [3] and acrolein [4], found in mustard oil and cigarette smoke, respectively. Endogenous TRPA1 activators are produced in inflammatory reactions, including reactive oxygen and nitrogen species [5–8]. More recently, TRPA1 has been found to be expressed also in non-neuronal cells, including chondrocytes [9], keratinocytes [10–12] and lung epithelial cells [13–15], and to regulate the expression of inflammatory factors such as interleukin 8 (IL-8), interleukin 6 (IL-6) and prostaglandin E_2_ [9, 13–23].

TRPA1 seems to be important in the pathogenesis of inflammatory lung diseases. TRPA1 is activated by cigarette smoke to cause inflammation and hyperreactivity [4, 17, 19], epithelial cell damage [17, 19] and emphysema [24]. In the ovalbumin model of allergic asthma, TRPA1 has been reported to promote the release of inflammatory factors [25–29], mediate peripheral blood eosinophilia [27, 29], increase leukocyte influx to the lungs [22, 25, 27–29] and to increase airway hyperreactivity [25, 26, 28, 29].

We and others have recently found that TRPA1 expression is upregulated by inflammatory factors in human cells. Examples include interleukin 1 beta (IL-1β) [9, 15], interleukin 1 alpha (IL-1α) [30], tumor necrosis factor alpha (TNF-α) [12, 15, 30], interferon gamma (IFN-γ) [15], interleukin 17 (IL-17), lipopolysaccharide (LPS) and resistin [9]. In addition, TRPA1 translocation to the plasma membrane can be induced by inflammatory factors. These include the cytokines TNF-α [31, 32] and IL-1α [33] and the second messengers protein kinase A (PKA) and phospholipase C (PLC) [34]. Inflammatory factors can also modulate TRPA1 channel function. Activation of bradykinin receptors can activate or potentiate TRPA1 in a PKA and PLC-dependent manner [35–37]. PLC also mediates protease-activated receptor 2-dependent TRPA1 potentiation [36] and TRPA1 activation by the cytokine thymic stromal lymphopoietin [38]. TRPA1 channel function may also be enhanced by the inflammatory factors TNF-α [21] and nerve growth factor (NGF) [39]. Some anti-inflammatory drugs have been shown to downregulate *TRPA1* expression, examples being the glucocorticoid dexamethasone [12, 15, 40], the antirheumatic drug aurothiomalate [40], and calcineurin inhibitors [12].

CD4^+^ T helper (Th) lymphocytes specialize into different effector subsets. Th1 cells produce IFN-γ and promote cell-mediated immunity–whereas the Th2 cells produce IL-4, promote immunity against parasites and have a role in allergy and humoral immunity. Several other Th subtypes have also been established recently (reviewed in [41, 42]). The Th1-Th2 paradigm is relevant when considering asthma which is divided into phenotypes and endotypes characterized by features of Th2 or non-Th2 type inflammation. [43–45].

We have previously found that the signature Th1 cytokine IFN-γ upregulates TRPA1 expression in human A549 lung epithelial cells exposed to inflammatory stimuli (TNF-α and IL-1β) [15]. However, the role of Th2 cytokines on TRPA1 expression remains unknown. As the Th1-Th2 paradigm is essential when considering pathogenesis of inflammatory diseases including asthma, we aimed to study the regulation of TRPA1 expression and function under Th1 and Th2-type inflammation in human A549 lung epithelial cells.

## Methods

### Cell culture

A549 alveolar epithelial cells (American Type Culture Collection, Manassas, VA, USA) were cultured in Ham’s F–12 K (Kaighn’s modification) medium with 10% heat-inactivated fetal bovine serum, 100 μg/ml streptomycin, 100 U/ml penicillin and 250 ng/ml amphotericin B (all from Gibco/Life Technologies, Carlsbad, CA, USA) at 37 ^◦^C in 5% CO_2_. A549 cells were seeded on 24-well plates and grown for 48 h before the experiments. During the experiments the cells were cultured with the following compounds or their combinations as indicated: TNF-α, IL-1β, IFN-γ, IL-4, IL-13 (all from R&D Systems Europe Ltd, Abingdon, UK), the Janus kinase (JAK) inhibitors bariticinib and tofacitinib, the STAT6 inhibitor AS1517499, the TRPA1 agonist allyl isothiocyanate (AITC) and the TRPA1 antagonists HC-030031 and A-967079 (all from Sigma Aldrich, St. Louis, MO, USA).

### RNA extraction and RT-PCR

Total RNA was extracted (GenElute Mammalian Total RNA Miniprep kit, Sigma Aldrich) at indicated time points, and was reverse-transcribed to cDNA (TaqMan^Ⓡ^ Reverse Transcription Reagents, Applied Biosystems, Foster City, CA, USA). PCR was carried out by using the Applied Biosystems 7500 Real-Time PCR instrument and Taqman Universal PCR Master Mix reagent. The primer and probe sequences and concentrations for *GAPDH* were designed and optimized using Primer Express software (Applied Biosystems) and were: 5′-AAGGTCGGAGTCAACGGATTT-3’ (*GAPDH*, forward, 300 nM), 5′-GCAACAATATCCACTTTACCAGAGTTAA-3’ (*GAPDH*, reverse, 300 nM), and 5′-CGCCTGGTCACCAGGGCTGC-3’ (*GAPDH*, probe, 150 nM, containing 6-FAM as 5′-reporter dye and TAMRA as 3′-quencher) (Metabion, Martinsried, Germany). TaqMan Gene Expression assay for TRPA1 (Hs00175798_m1) was obtained from Life Technologies (Life Technologies Europe BV, Bleiswijk, the Netherlands). In data analysis, mRNA expression levels were first normalized against *GAPDH* mRNA levels, and the ΔΔCt method was used in the calculations.

### Western blot

Protein extraction, TRPA1 immunoprecipitation and Western blot analysis were carried out as described previously [9, 12] with slight modifications. In this study, each sample containing 1950 µg of total protein was subjected to immunoprecipitation. As previously, the TRPA1 antibody SAB2105082 (Sigma Aldrich) and Protein A/G PLUS-Agarose (sc-2003, Santa Cruz Biotechnology, Inc., Dallas, TX, USA) were used in TRPA1 immunoprecipitation. In the Western blot analysis, NB110-40,763 (Novus Biologicals, LCC, Littleton, CO, USA) diluted in 1:1000 in 5% non-fat milk was used as the primary antibody. Goat anti-rabbit HRP-linked IgG antibody CST#7074 diluted in 1:10 000 in 5% non-fat milk (Cell Signaling Technology Inc., Beverly, MA, USA) was used as the secondary antibody. HEK293 cells (American Type Culture Collection, Manassas, VA, USA) transfected with TRPA1 plasmid DNA (pCMV6-XL4 by Origene, Rockville, MD, USA) were used as a positive control. HEK293 culturing and transfection were carried out as described previously [9, 12].

### Immunoassay

Lipocalin-2 (LCN2) and chemokine (C-X-C motif) ligand 6 (CXCL6) concentrations in A549 medium samples were measured by enzyme-linked immunosorbent assay (ELISA). The reagents were purchased from R&D Systems Europe Ltd, Abingdon, United Kingdom.

### Intracellular Ca^2+^ measurements

TRPA1-dependent changes in intracellular Ca^2+^ levels were determined using the fluo-3-acetoxymethyl ester assay (Fluo 3-AM, Sigma Aldrich) as described previously [46]. In brief, after culturing the A549 cells in indicated experimental conditions, the cells were loaded in room temperature with 4 μM Fluo 3-AM and 0.08% Pluronic F-127^Ⓡ^ in Hanks’ balanced salt solution (HBSS, Lonza, Verviers, Belgium) containing 1 mg/ml bovine serum albumin, 2.5 mM probenecid and 25 mM HEPES pH 7.2 (all from Sigma Aldrich) for 30 min. The excitation/emission wavelengths of 485/535 nm were analyzed using Victor3 1420 multilabel counter (PerkinElmer, Waltham, MA, USA) as an indicator of free intracellular Ca^2+^. The cells were first preincubated for 30 min at room temperature with the TRPA1 antagonist HC-030031 (200 μM, Sigma Aldrich) or the vehicle (DMSO). Thereafter, using an injector, the TRPA1 agonist allyl isothiocyanate (AITC, 100 μM, Sigma Aldrich) was applied and the measurements were continued for 30 s.

### Statistical analysis

Statistical analysis was performed using Graph-Pad Prism version 5.02 (GraphPad Software, San Diego, CA, USA). The results are presented as mean ± standard error of the mean (SEM). One-way analysis of variance (ANOVA) or repeated measures ANOVA followed by Bonferroni’s multiple comparisons test were used as indicated.

## Results

### The effect of Th1 and Th2 cytokines on TRPA1 expression in inflammatory conditions

**Fig. 1:**
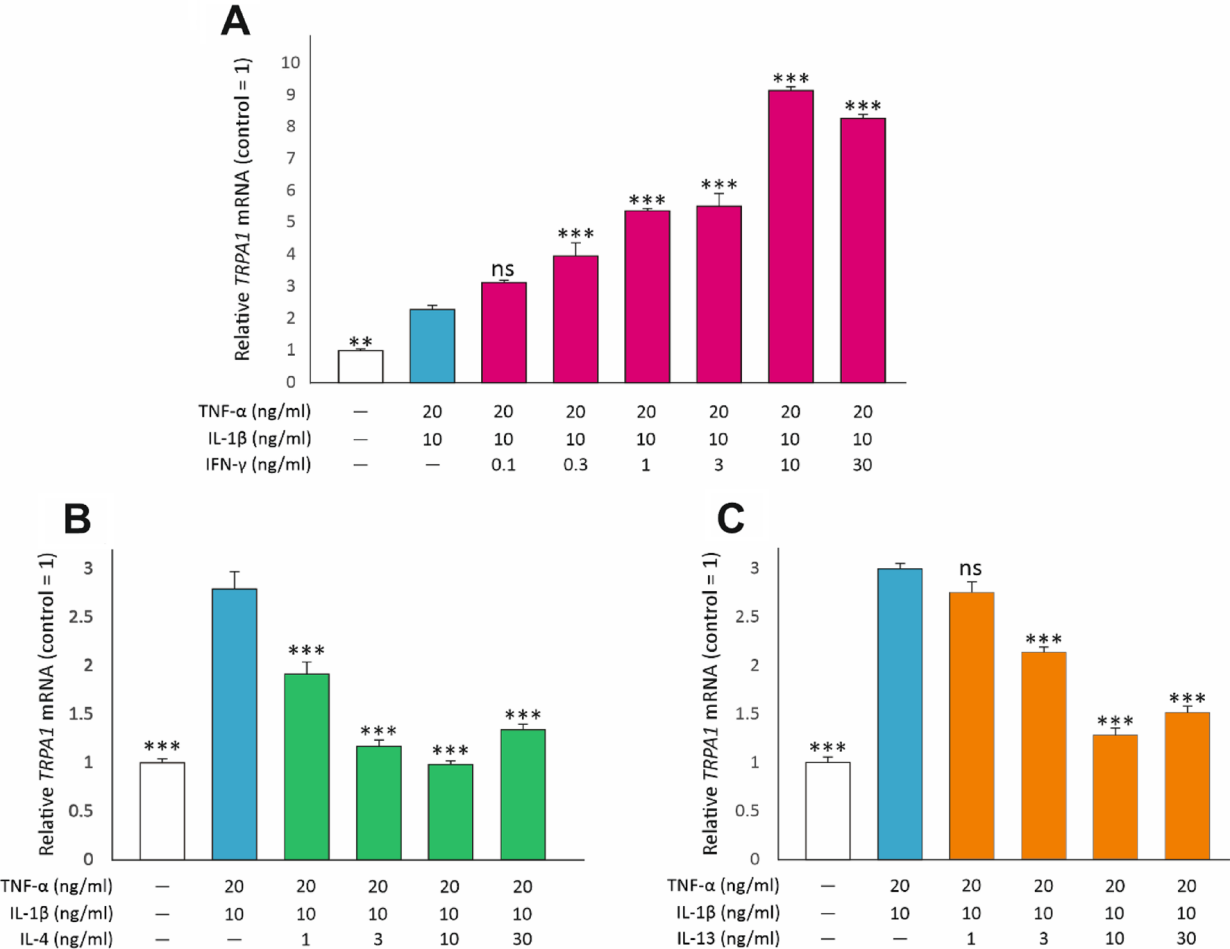
The Th1 cytokine IFN-γ upregulates and the Th2 cytokines IL-4 and IL-13 downregulate *TRPA1* expression. In **(A)**, **(B)** and **(C)**, the dose-dependent effect of the cytokines is shown. A549 cells were cultured for 24 h with the combination of TNF-α (20 ng/ml) and IL-1β (10 ng/ml), and increasing concentrations of IFN-γ **A**, IL-4 **B** or IL-13 **C**. Thereafter, total RNA was extracted and *TRPA1* RT-PCR was performed against *GAPDH* as a housekeeping gene. In all figures, data is presented as mean + SEM, *n* = 4. Comparisons were carried out using One-way ANOVA with Bonferroni’s post-test. Asterisks on bars indicate comparison against the combination of TNF-α and IL-1β. ** and *** denote *p* < 0.01 and *p* < 0.001, respectively; ns = not significant

TRPA1 expression was low in dormant A549 lung epithelial cells but it was significantly increased in inflammatory conditions when the cells were cultured with the combination of TNF-α and IL-1β. When the classical Th1 cytokine IFN-γ was added to the culture, it further enhanced TRPA1 expression. The effect of IFN-γ was statistically significant already at 0.3 ng/ml concentration and increased in a dose-dependent manner up to 10 ng/ml concentration. In contrast, the Th2 cytokines IL-4 and IL-13 significantly downregulated TNF-α and IL-1β-induced *TRPA1* expression. The effect was dose-dependent and the concentration of 10 ng/ml reduced *TRPA1* expression to the control level. (Fig. 1) Based on the concentration curves, we chose to continue experiments with the concentration of 10 ng/ml of IFN-γ, IL-4 and IL-13.

**Fig. 2:**
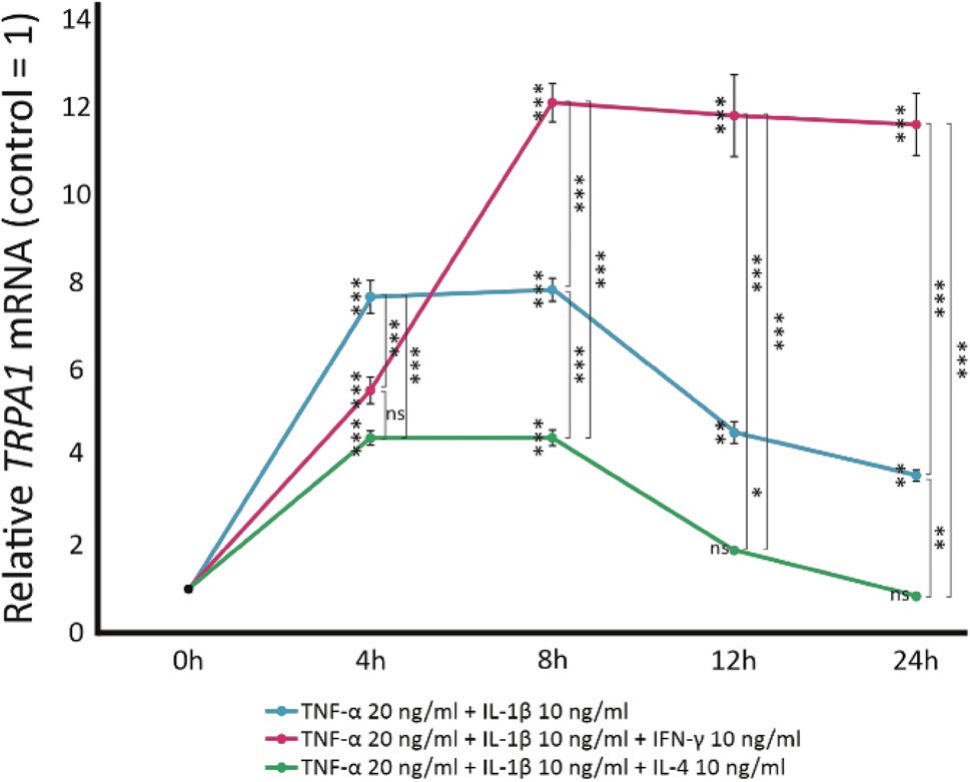
The time-dependent effects of Th1 and Th2-type cytokines on *TRPA1* mRNA expression. A549 cells were cultured with the combination of TNF-α (20 ng/ml) and IL-1β (10 ng/ml), with and without IFN-γ (10 ng/ml) or IL-4 (10 ng/ml). At 0, 4, 8, 12 and 24 h, total RNA was extracted and *TRPA1* RT-PCR was performed against *GAPDH* as a housekeeping gene. Control of each time point is set as 1, and the other values of the same time point are given in relation to that value. Mean ± SEM, *n* = 4. Comparisons were carried out using one-way ANOVA with Bonferroni’s post-test. Asterisks beside the data points represent comparisons against the control (untreated sample) of the respective time point, and asterisks beside brackets represent comparisons carried out between indicated conditions. *, ** and *** denote *p* < 0.05, < 0.01 or < 0.001, respectively; ns = not significant

In Fig. 2, the time curves of the effects of IFN-γ and IL-4 are shown. When the cells were first exposed to the combination of TNF-α and IL-1β, it was found to induce an increase in *TRPA1* expression reaching its peak at four and eight hours and thereafter declining. When IFN-γ was added into the culture, it further enhanced *TRPA1* expression which was obvious at eight hours and thereafter remained stable up to the 24 h follow-up being at about 12 folds higher level compared to the control expression. Adding IL-4 to the culture significantly downregulated TNF-α and IL-1β-induced *TRPA1* expression at all time points, reaching control level at 12–24 h.

**Fig. 3:**
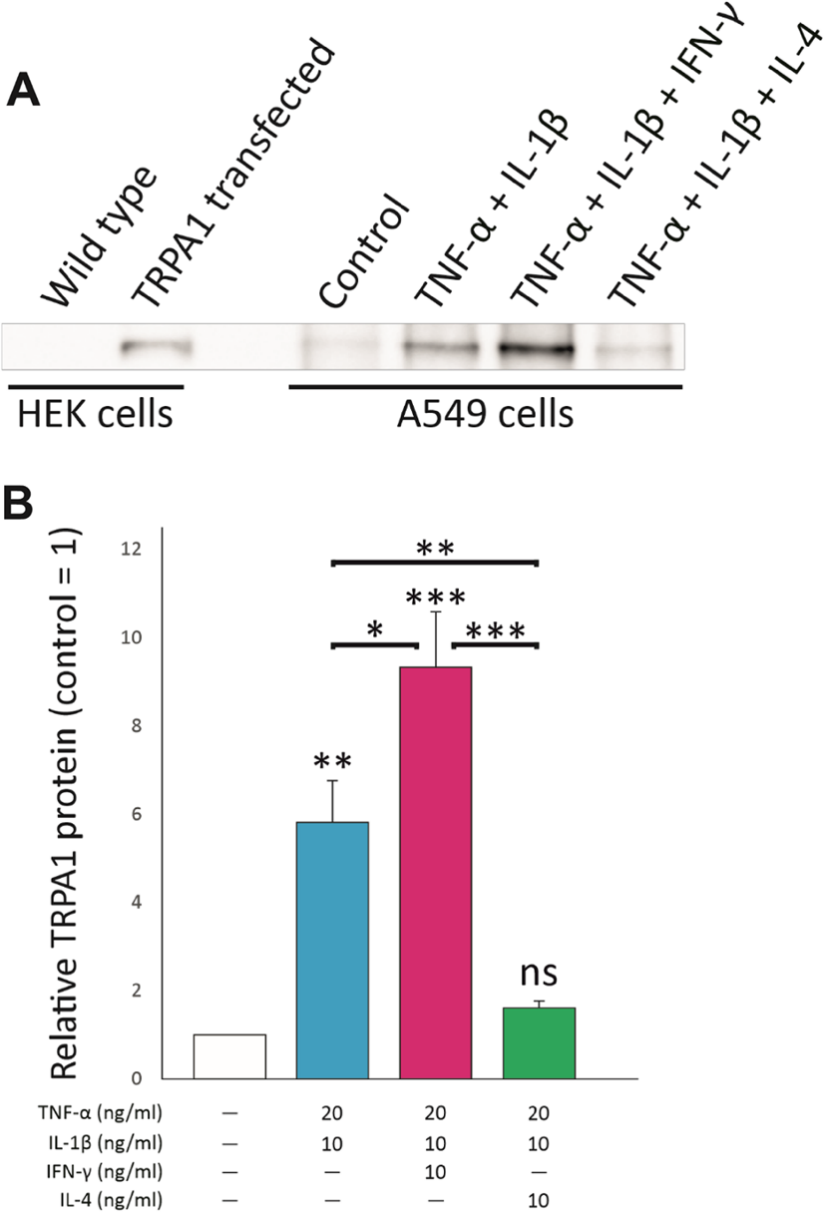
The effects of Th1 and Th2 cytokines on TRPA1 protein expression under inflammatory conditions. A549 cells were cultured with the combination of TNF-α (20 ng/ml) and IL-1β (10 ng/ml), with and without IFN-γ (10 ng/ml) or IL-4 (10 ng/ml) for 12 h. Thereafter TRPA1 protein levels were measured with Western blot after immunoprecipitation. TRPA1-transfected HEK293 cells were used as a positive control. In **A** is shown a representative immunoblot (a complete gel profile is shown in the Supplementary Figure S1), and in **B** a densitometric analysis of five independent experiments. In each experiment, the densitometric signal in the control was set as 1 and the other values are given in relation to that value. The data is presented as mean + SEM. Statistical analysis was performed by using repeated measures ANOVA with Bonferroni’s post-test. Asterisks on bars indicate comparison to control, and asterisks on brackets indicate comparison between depicted conditions. *, ** and *** denote p < 0.05, < 0.01 and 0.001, respectively. Ns = not significant

We then studied whether the changes in *TRPA1* mRNA also correlate to TRPA1 protein levels. A549 cells were cultured in same conditions as above, protein extraction was carried out and TRPA1 was measured by Western blot after immunoprecipitation. As expected, the combination of TNF-α and IL-1β significantly upregulated TRPA1 protein expression. In accordance with the mRNA data, combining IFN-γ to the treatment further enhanced TRPA1 protein levels; whereas IL-4 significantly downregulated TNF-α and IL-1β-induced TRPA1 protein expression which did not differ from the control level (Fig. 3).

**Fig. 4:**
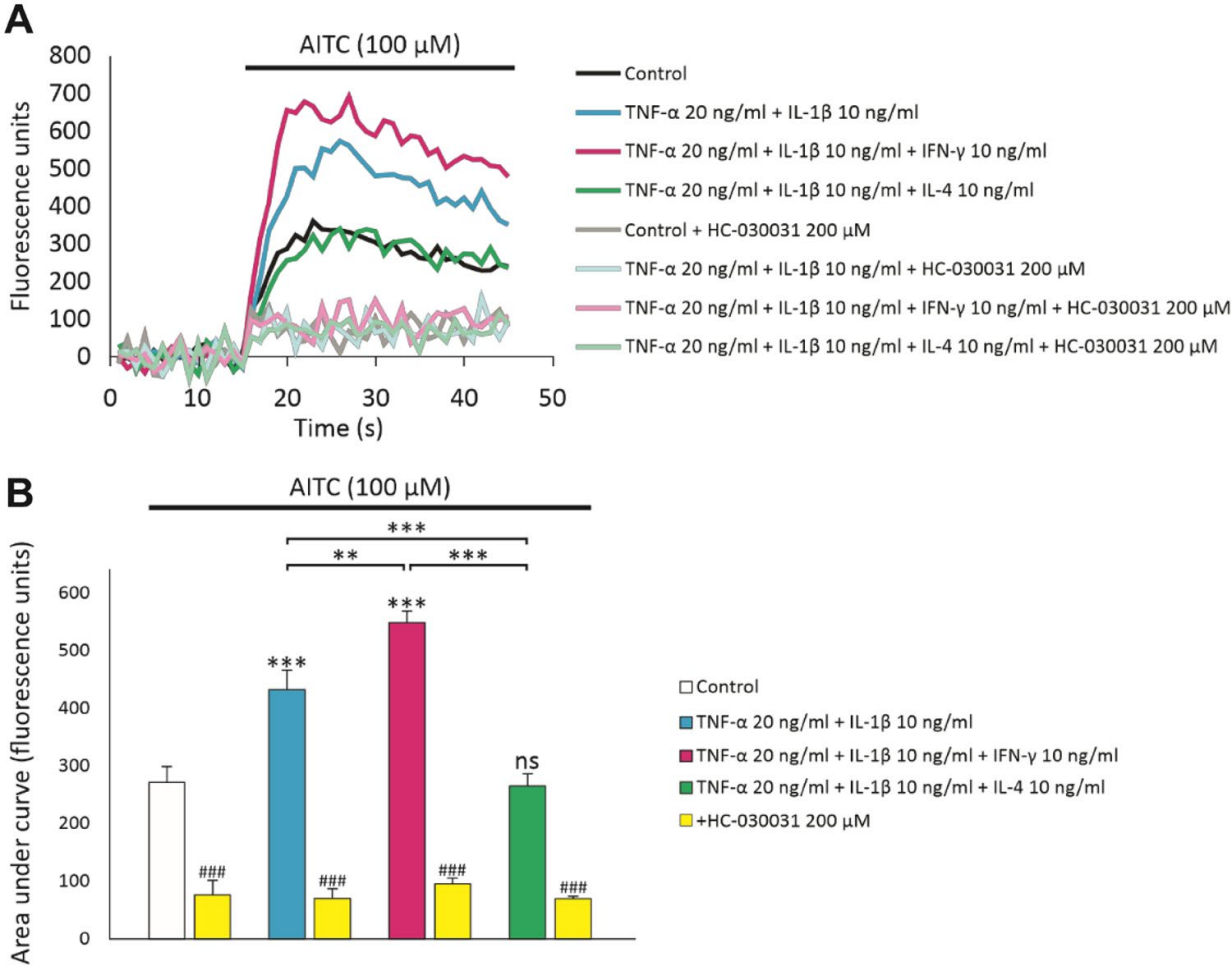
The effects of inflammation as well as Th1 and Th2 conditions on TRPA1 function. Prior to the intracellular Ca^2+^ measurements, A549 cells were cultured with the combination of TNF-α (20 ng/ml) and IL-1β (10 ng/ml), with or without IFN-γ (10 ng/ml) or IL-4 (10 ng/ml) for 48 h. Thereafter, the cells were loaded with the calcium-responsive fluorescent dye Fluo 3-AM, and the cells were preincubated with the TRPA1 antagonist HC-030031 (200 µM) or vehicle (DMSO) for 30 min. After measuring baseline fluorescence, the TRPA1 agonist allyl isothiocyanate (AITC, 100 µM) was applied and fluorescence was measured for 30 s. In **A** is shown the averaged trace from 8 wells and in **B** is shown the area under curve analysis as mean + SEM, *n *= 8. Statistical analysis was performed using one-way ANOVA with Bonferroni’s post-test. Asterisks on bars indicate comparison against control, asterisks on brackets indicate comparison between depicted conditions; and hashtags on bars indicate comparison against the other bar of the respective condition. **, *** and ### denote *p* < 0.01, *p* < 0.001 and *p* < 0.001, respectively; ns = not significant

Next, we investigated whether the observed differences in TRPA1 mRNA and protein levels translate into differences in TRPA1 function and utilized the Fluo 3-AM intracellular calcium assay. A549 cells were cultured in the same conditions as above. In all conditions, the TRPA1 agonist allyl isothiocyanate (AITC) induced a fluorescent signal, indicating that intracellular Ca^2+^ concentration was increased. These effects were reversed by the TRPA1 antagonist HC-030031, indicating the presence of functional TRPA1 channel. In cells cultured in the presence of the combination of TNF-α and IL-1β, the Ca^2+^ signal was significantly increased as compared to controls. When IFN-γ was added to the culture, the Ca^2+^ signal further increased, whereas added IL-4 decreased the signal to the control level (Fig. 4). These data indicate that the enhancing effect of the Th1 cytokine IFN-γ and the decreasing effect of the Th2 cytokine IL-4 on TRPA1 is present at mRNA, protein and functional level.

### The effect of Janus kinase (JAK) inhibitors on IFN-γ and IL-4-induced changes in *TRPA1* expression

**Fig. 5:**
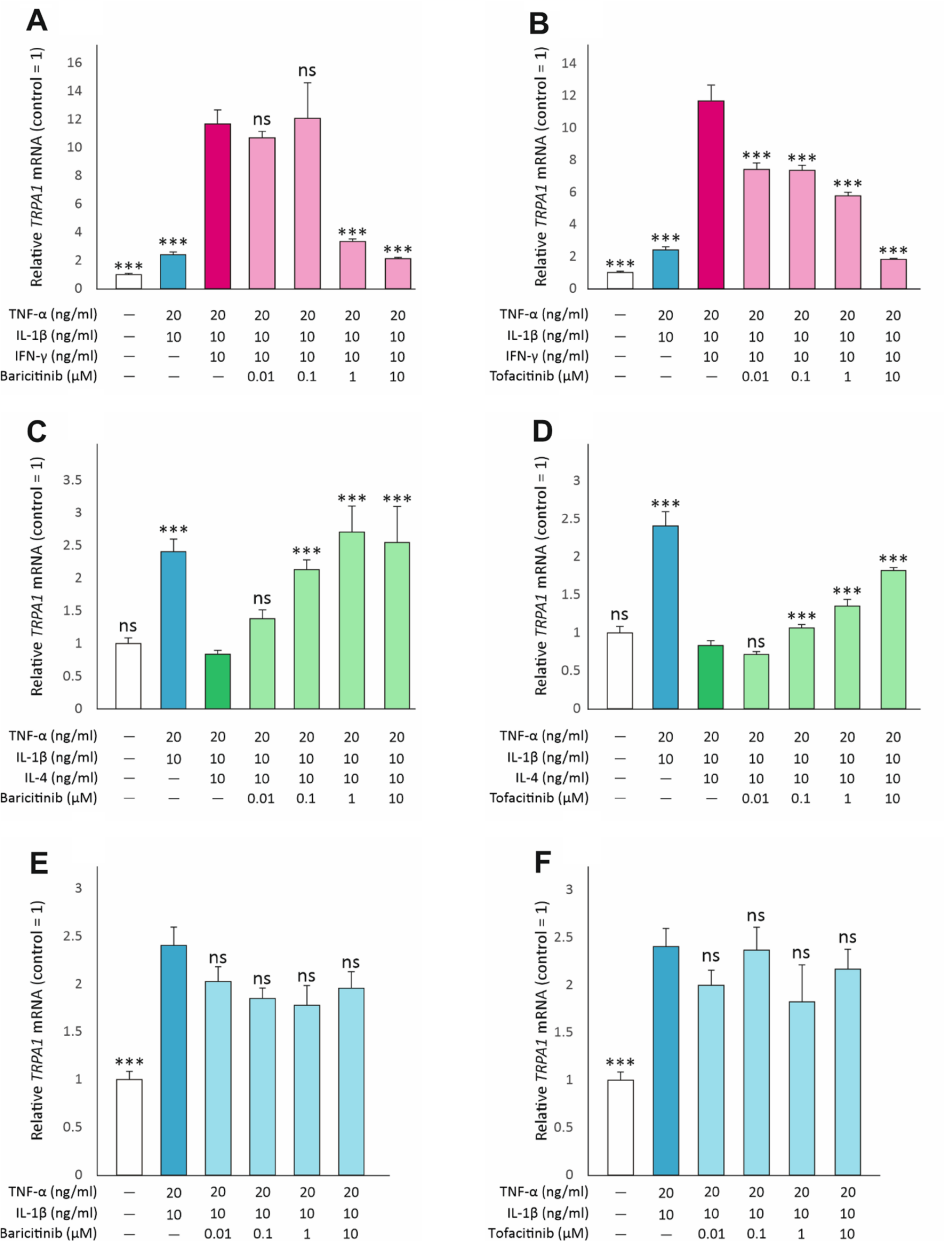
JAK inhibitors reverse the effects of IFN-γ and IL-4 on *TRPA1* expression. A549 cells were cultured for 24 h with the combination of TNF-α (20 ng/ml) and IL-1β (10 ng/ml), with or without IFN-γ (10 ng/ml; **A** and **B**) or IL-4 (10 ng/ml; **C** and **D**) and increasing concentrations of baricitinib (0.01–10 µM; **A**, **C** and** E**) or tofacitinib (0.01-10 µM; **B**, **D** and** F**). Thereafter, total RNA was extracted and *TRPA1* mRNA expression was measured by RT-PCR against *GAPDH* as a housekeeping gene. Data is presented as mean + SEM, n = 4–8. Comparisons were carried out using one-way ANOVA with Bonferroni’s post-test. Asterisks on bars indicate comparison to the treatment with TNF-α + IL-1β + IFN-γ **(A,B)** or TNF-α + IL-1β + IL-4 **(C,D)**. *** denotes *p* < 0.001, ns = not significant

In their target cells, IFN-γ and IL-4 activate Janus kinase (JAK)—signal transducer and activator of transcription (STAT) signaling pathways which mediate many of their cellular effects [47, 48]. Therefore, we investigated the effects of two JAK inhibitors, baricitinib and tofacitinib, on the IFN-γ and IL-4 induced changes in TRPA1 expression. Baricitinib and tofacitinib at concentrations of 0.1—10 µM significantly reversed the effects of IFN-γ and IL-4 on TRPA1 expression but did not affect TRPA1 expression induced by TNF-α and IL-1β in the absence of IFN-γ and IL-4. (Fig. 5).

**Fig. 6:**
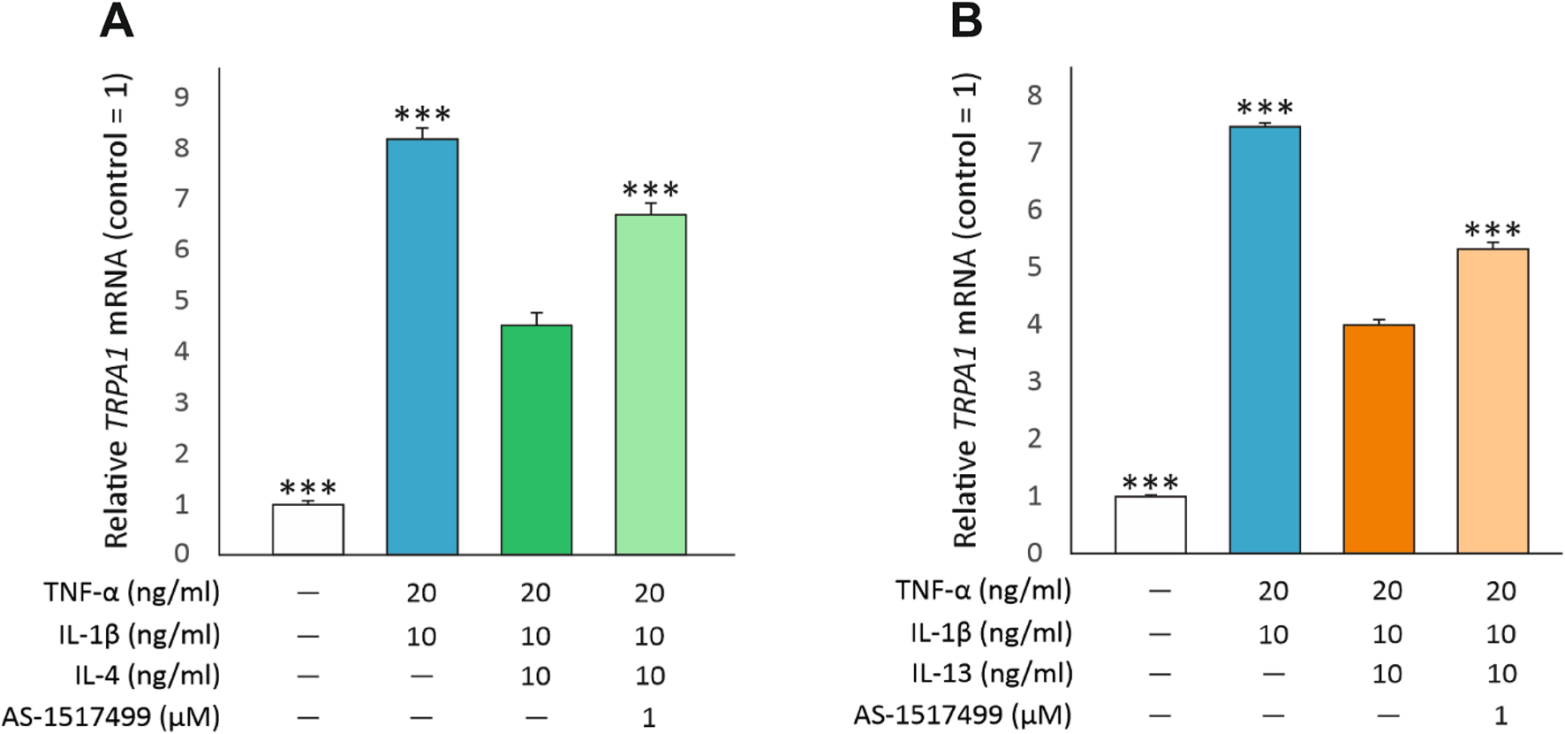
STAT6 inhibitor partly reverses the effects of IL-4 and IL-13 on *TRPA1* expression. A549 cells were cultured with the combination of TNF-α (20 ng/ml) and IL-1β (10 ng/ml), with or without IL-4 (10 ng/ml, **A**) or IL-13 (10 ng/ml, **B**) and the STAT6 inhibitor AS1517499 (1 µM) for 8 h. Thereafter, total RNA was extracted and *TRPA1* mRNA was measured by RT-PCR against *GAPDH* as a housekeeping gene. Data is presented as mean + SEM, *n* = 4. Comparisons were carried out using one-way ANOVA with Bonferroni’s post-test. Asterisks on bars indicate comparison to the treatment with TNF-α + IL-1β + IL-4 **(A)** or TNF-α + IL-1β + IL-13 **(B)**. *** denotes *p *< 0.001

JAK signaling activated by IL-4 and IL-13 leads to the phosphorylation and translocation of STAT6 whereas IFN-γ activates primarily STAT1 [47, 48]. Accordingly, the STAT6 inhibitor AS1517499 [49] reversed the downregulation of *TRPA1* expression induced by IL-4 and IL-13 (Fig. 6).

### The effects of the anti-inflammatory drugs dexamethasone and rolipram on *TRPA1* expression

**Fig. 7:**
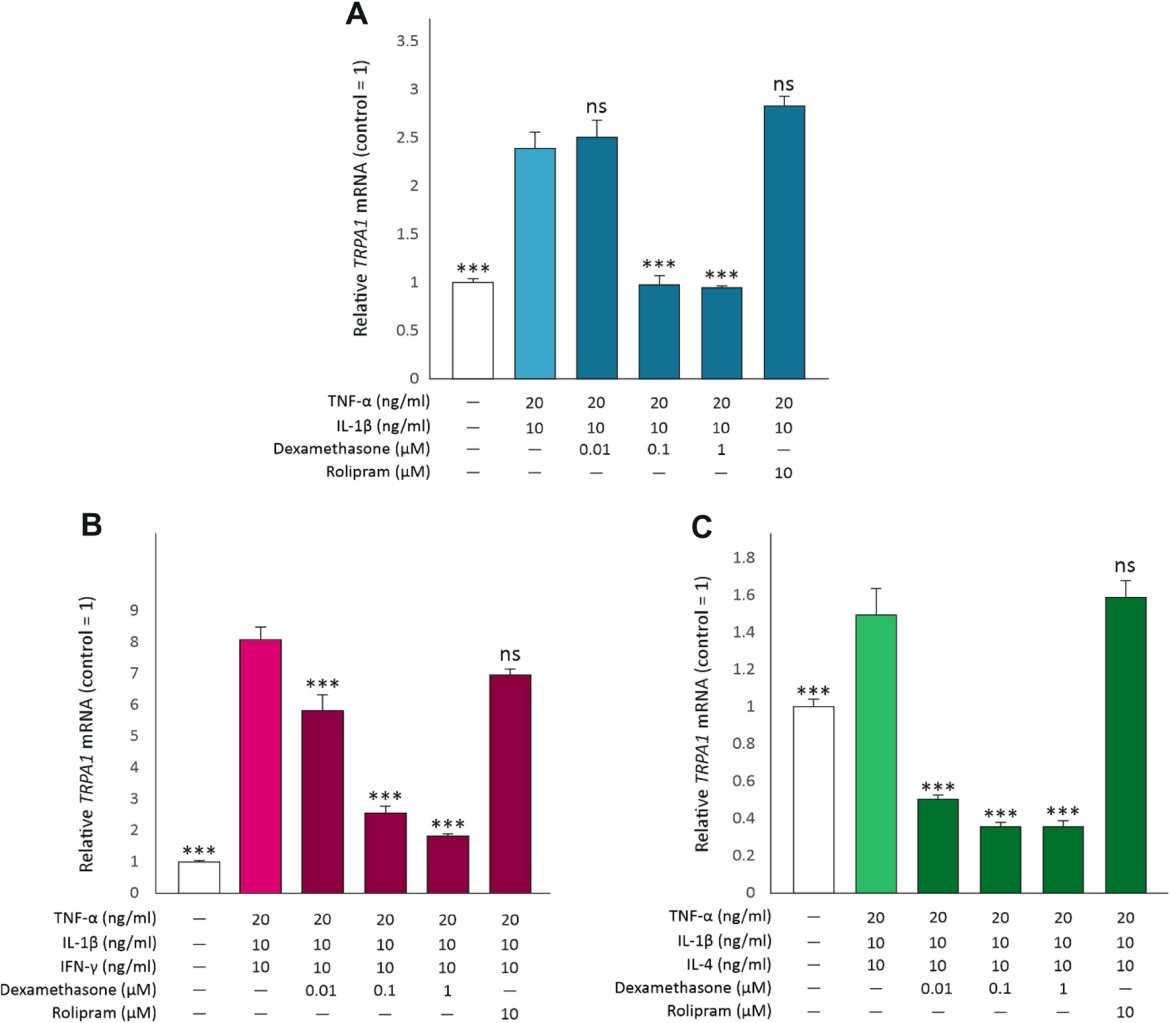
Dexamethasone downregulates *TRPA1* but rolipram has no effect. A549 cells were cultured for 24 h with the combination of TNF-α (20 ng/ml) and IL-1β (10 ng/ml, **A**), with or without IFN-γ (10 ng/ml, **B**) or IL-4 (10 ng/ml, **C**), and rolipram (10 µM) or increasing concentrations of dexamethasone (0.01–1 µM). Thereafter, total RNA was extracted and *TRPA1* mRNA was measured using RT-PCR against *GAPDH* as a housekeeping gene. Data is presented as mean + SEM, n = 4. Comparisons were carried out using one-way ANOVA with Bonferroni’s post-test. Asterisks on bars indicate comparison to the treatment with TNF-α + IL-1β (**A**), TNF-α + IL-1β + IFN-γ (**B**) or TNF-α + IL-1β + IL-4 (**C**). *** denotes *p* < 0.001, ns = not significant

Glucocorticoids and PDE4 inhibitors are used as anti-inflammatory treatments in lung diseases. Therefore, we investigated the effects of the glucocorticoid dexamethasone and the PDE4 inhibitor rolipram on *TRPA1* expression. Dexamethasone downregulated *TRPA1* expression in a dose-dependent manner independent on whether the cells were cultured with TNF-α + IL-1β only or in a combination with IFN-γ or IL-4. Full effect was achieved at 0.1 µM drug concentration in the case of TNF-α + IL-1β and TNF-α + IL-1β + IL-4 treated cells, whereas 1 µM was required when the cells were treated with a combination of TNF-α + IL-1β + IFN-γ. In contrast, rolipram had no effect on *TRPA1* expression (Fig. 7).

### The effect of TRPA1 on lipocalin-2 (LCN2) and chemokine (C-X-C motif) ligand 6 (CXCL6) expression

**Fig. 8:**
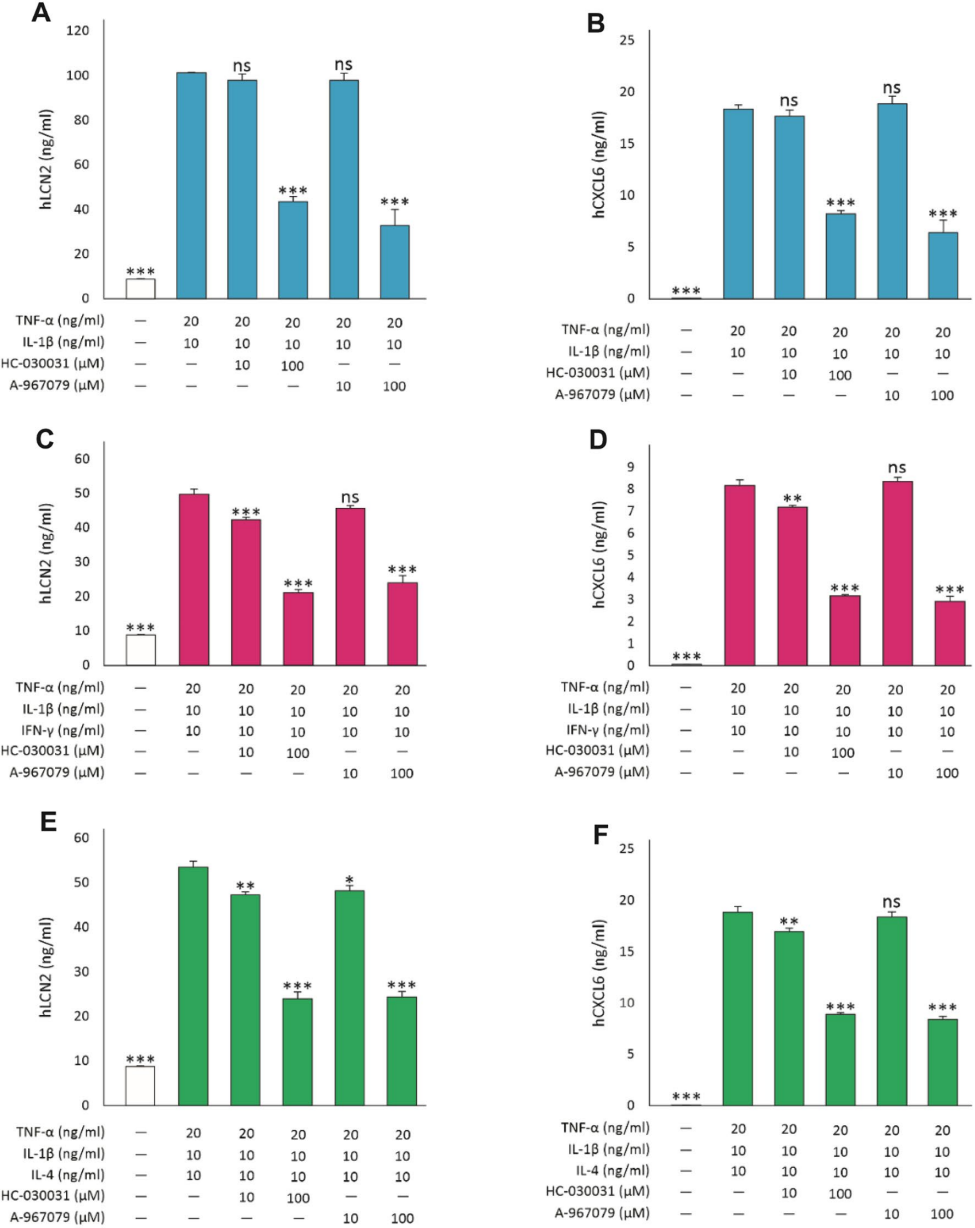
TRPA1 regulates lipocalin-2 (LCN2) and chemokine (C-X-C motif) ligand 6 (CXCL6) production. Left: LCN2; right: CXCL6. A549 cells were cultured with the combination of TNF-α (20 ng/ml) and IL-1β (10 ng/ml) **(A,B)**, with IFN-γ (10 ng/ml**; C,D)** or IL-4 (10 ng/ml; **E,F)**; and the TRPA1 antagonists HC-030031 (10 and 100 µM) and A-967079 (10 and 100 µM) for 24 h. Thereafter, culture medium was collected and enzyme-linked immunosorbent assay (ELISA) was applied. The data is presented as mean + SEM, *n* = 3–4. Statistical analysis was performed using one-way ANOVA with Bonferroni’s post-test. Asterisks on bars indicate comparison against the stimulated condition without inhibitors. *, ** and *** denote *p* < 0.05, < 0.01 and 0.001, respectively. ns = not significant

We were interested in investigating if TRPA1 regulates the expression of inflammatory genes in lung epithelial cells. Based on our preliminary RNA-sequencing data, we chose to focus on lipocalin-2 (LCN2) and chemokine (C-X-C motif) ligand 6 (CXCL6). The production of LCN2 and CXCL6 was increased when A549 cells were cultured in the presence of the combination of TNF-α and IL-1β, with or without IFN-γ or IL-4. TRPA1 inhibitors HC-030031 and A-967079 significantly reduced the production of LCN2 and CXCL6 in all conditions, indicating that TRPA1 enhances the production of these factors in A549 cells (Fig. 8).

## Discussion

The present study shows that TRPA1 expression and function in human lung epithelial cells is regulated by inflammation and Th1 and Th2-type cytokines and that TRPA1 modulates gene expression relevant in innate immunity and inflammation. We demonstrate that inflammatory conditions (depicted by adding TNF-α and IL-1β into the culture) enhance TRPA1 expression and function, and that the Th1 cytokine IFN-γ further upregulates TRPA1. For the first time, we show that the Th2-type cytokines IL-4 and IL-13 significantly downregulate TRPA1 expression and function. Additionally, IFN-γ and IL-4 effects were shown to be reversed by JAK inhibitors; and the effects of IL-4 and IL-13 to be at least partially dependent on the transcription factor STAT6. Moreover, the expression of LCN2 and CXCL6 was suppressed by pharmacological TRPA1 blockade, which is also novel.

Early work showed that in the human airways, TRPA1 is expressed in epithelial cells, smooth muscle cells and fibroblasts, and promotes inflammatory responses [13, 14]. Thereafter, inflammatory stimuli have been shown to upregulate TRPA1. In A549 lung epithelial cells, IL-1α was reported to sensitize TRPA1 function by increasing TRPA1 translocation to the plasma membrane [33]. In lung fibroblasts, TNF-α has been shown to sensitize TRPA1 function [21]. Our recent work [15] showed that in A549 cells, combinations of inflammatory cytokines upregulate TRPA1 expression. The present study strengthens the view of TRPA1 as an inflammation-increased factor, as we show that the combination of TNF-α and IL-1β enhances TRPA1 expression on mRNA and protein levels and significantly increases TRPA1 function.

In our view, however, the most significant novel finding of this study is the striking difference between the effects of Th1 and Th2-type cytokines on TRPA1 expression and function in the lung epithelial cells: the Th1 type cytokine IFN-γ upregulated, while the Th2 type cytokine IL-4 downregulated TRPA1 expression and function. In support to this, we confirmed that the effects of IFN-γ and IL-4 are JAK-dependent as they were reversed by the JAK inhibitors baricitinib and tofacitinib. In addition, the effects of IL-4 and IL-13 seemed to be STAT6-dependent.

Our findings suggest that in lung epithelium TRPA1 is upregulated under inflammation and further enhanced under Th1-type inflammatory conditions while it is downregulated towards normal levels in Th2-type conditions. This may have meaningful implications in inflammatory (lung) diseases. For instance, asthma is expressed in various phenotypes. Allergic asthma is an example of the Th2-related phenotype, whereas non-Th2 asthma includes for example smoking associated and obesity associated asthma [43–45]. TRPA1 has been shown to mediate asthmatic inflammation and hyperresponsiveness in models of allergic asthma [25–29]. Therefore, TRPA1 has attracted attention as a potential asthma drug target. Our results show that TRPA1 function in inflamed lung epithelium could be more pronounced in Th1-type inflammation than in Th2-type conditions. This could further influence the feasibility of targeting TRPA1 in lung inflammation. One could expect relatively greater TRPA1 function and therefore a more pronounced response to TRPA1 antagonists in Th1-type conditions, such as in viral infection and perhaps non-Th2 asthma. Conversely, in Th2-type conditions–such as in allergic asthma–response to TRPA1 antagonist therapy might be more limited, as the channel would already be moderately downregulated/desensitized. Considering this, targeting TRPA1 could be more effective in non-Th2 asthma. It is also possible that the suppressive effect of IL-4 on the functional expression of TRPA1 is a compensation mechanism aiming to limit inflammation and symptoms in allergic asthma.

However, in addition to non-Th2 asthma, targeting TRPA1 in Th2 asthma could still be a viable strategy. While in our A549 model TNF-α and IL-1β induced TRPA1 expression was downregulated by IL-4, it remained functional in the Fluo 3-AM assay and was able to regulate CXCL6 and LCN2 expression. This suggests that the remaining TRPA1 function could be sufficient to carry out biologically relevant functions also under IL-4 stimulation and could possibly be involved in the development of asthmatic responses. However, inflammation ultimately is a vastly complex in vivo response and therefore the data from our in vitro model cannot be extrapolated directly to the in vivo scenario. As such, further studies are needed to confirm these effects in vivo.

Glucocorticoids and PDE4 inhibitors are used as anti-inflammatory treatments in lung diseases. In the present study we found that dexamethasone downregulated *TRPA1* expression under all conditions tested. This is supported by our previous results showing that dexamethasone downregulated *TRPA1* expression in chondrocytes, keratinocytes and lung epithelial cells under inflammatory conditions [12, 15, 40]. The full effect was achieved at rather small concentrations (0.1–1 µM). These results indicate that *TRPA1* expression is sensitive to glucocorticoid treatment and that the effect is likely present in different types of inflammation. The results also suggest that *TRPA1* downregulation is an additional anti-inflammatory/analgesic mechanism of action of glucocorticoids.

We also examined the effect of the PDE4 inhibitor rolipram [50] on *TRPA1* expression. PDE4 is an intracellular enzyme catalyzing the hydrolysis of the major intracellular signaling molecule cyclic AMP (cAMP) to its inactive metabolites [51]. Therefore, inhibiting PDE4 results in increased intracellular cAMP levels following cell activation through various G-protein coupled receptors. This leads to the activation of protein kinase A and CREB transcription factor to modulate gene expression and inflammatory response [50]. The use of PDE4 inhibitors is approved in asthma and COPD [50]. In the present study we used the representative PDE4 inhibitor rolipram and did not observe a significant effect on *TRPA1* expression. These data suggest that PDE4 inhibitors–and therefore pathways responsive to cAMP concentration–do not likely control *TRPA1* expression in lung epithelial cells. However, we cannot exclude the possibility of an effect in some other conditions.

In the present study, the JAK inhibitors baricitinib and tofacitinib reversed the effects of IFN-γ and IL-4 on *TRPA1* expression in a dose-dependent manner. Baricitinib at 1 µM drug concentration was sufficient to produce a maximal effect whereas tofacitinib seems to be somewhat less potent. Binding of IFN-γ to its receptor leads to signaling through JAK1 and JAK2 [47] whereas binding of IL-4 to the type II IL-4 receptor (predominant receptor in the epithelium) leads to JAK1, TYK2 and JAK2 activation [48]. Baricitinib is considered a selective JAK1/2 inhibitor, whereas tofacitinib was originally developed as a JAK3 inhibitor but is also found to inhibit JAK1 [52]. The observed difference in the potency of the inhibitors could be explained by these differences in cytokine signaling and JAK selectivity of the inhibitors. Among other indications, baricitinib is recommended for the treatment of severe COVID-19 infection [53]. Our results imply that in the inflammatory environment in the lung (for example during COVID-19 infection), JAK inhibitor therapy could up- or downregulate TRPA1 expression depending on the Th1-Th2 balance.

TRPA1 has been shown to promote leukocyte infiltration to the lung, including neutrophils in models of airway inflammation [20, 22, 28]. This could be partially explained by the findings that TRPA1 promotes the production of the neutrophil-attracting chemokine interleukin 8 (IL-8) [13–16, 18–22]. In the present study, we found that TRPA1 antagonists in all tested conditions significantly reduced CXCL6 and LCN2 production as measured by ELISA. CXCL6 is a chemokine and binds the same receptors as IL-8 [54–57], which leads to neutrophil chemotaxis and activation. CXCL6 is also linked to pulmonary fibrosis and cystic fibrosis; it is upregulated in these conditions [58, 59] and its inhibition could have a beneficial effect on remodeling in asthmatic lungs. LCN2 is a secreted protein, essential in innate immunity [60] and sequesters iron [61]. LCN2 has chemoattractant and immunomodulatory properties, for example promoting neutrophil chemotaxis [62–64]. In addition, LCN2 inhibits the growth of certain bacteria [65, 66].

Our results suggest that TRPA1 in the lung epithelium promotes CXCL6 and LCN2 production and could therefore be important in the innate immunity and neutrophil chemotaxis. This could further have consequences when targeting TRPA1 for pharmacotherapy. Anti-inflammatory effects could be expected through reduced neutrophil infiltration in the lungs when TRPA1 is inhibited and CXCL6 and LCN2 subsequently downregulated. On the other hand, decreased LCN2 levels as well as decreased neutrophilia might increase susceptibility to bacterial infection.

## Conclusions

The present study reveals TRPA1 in the A549 model of lung epithelium as a factor regulated by Th1 and Th2-type inflammation: expressed and functioning predominantly under Th1-type inflammation and downregulated under Th2-type inflammation. We demonstrated the novel role of the JAKs and the transcription factor STAT6 in regulating *TRPA1* expression and showed *TRPA1* to be responsive to glucocorticoid treatment. We also discovered novel functions for TRPA1 in the lung epithelium in innate immunity and inflammation through regulating LCN2 and CXCL6 expression. We propose that the paradigm of Th1 and Th2-type inflammation is a major determinant of TRPA1 expression and function, and this should be considered when targeting TRPA1 for pharmacotherapy in (lung) disease.

## Supplementary Information

Below is the link to the electronic supplementary material.

Supplementary file1 (TIF 10270 KB)

## Data Availability

Data that support the findings and conclusions of this study are included within the article.
