# Supplementary figures and images for: The regulation of TRPA1 expression and function by Th1 and Th2-type inflammation in human A549 lung epithelial cells
Source: Inflamm Res. 2023 Jun 29;72(7):1327–39. doi: 10.1007/s00011-023-01750-y (PMC10352175; doi:10.1007/s00011-023-01750-y)

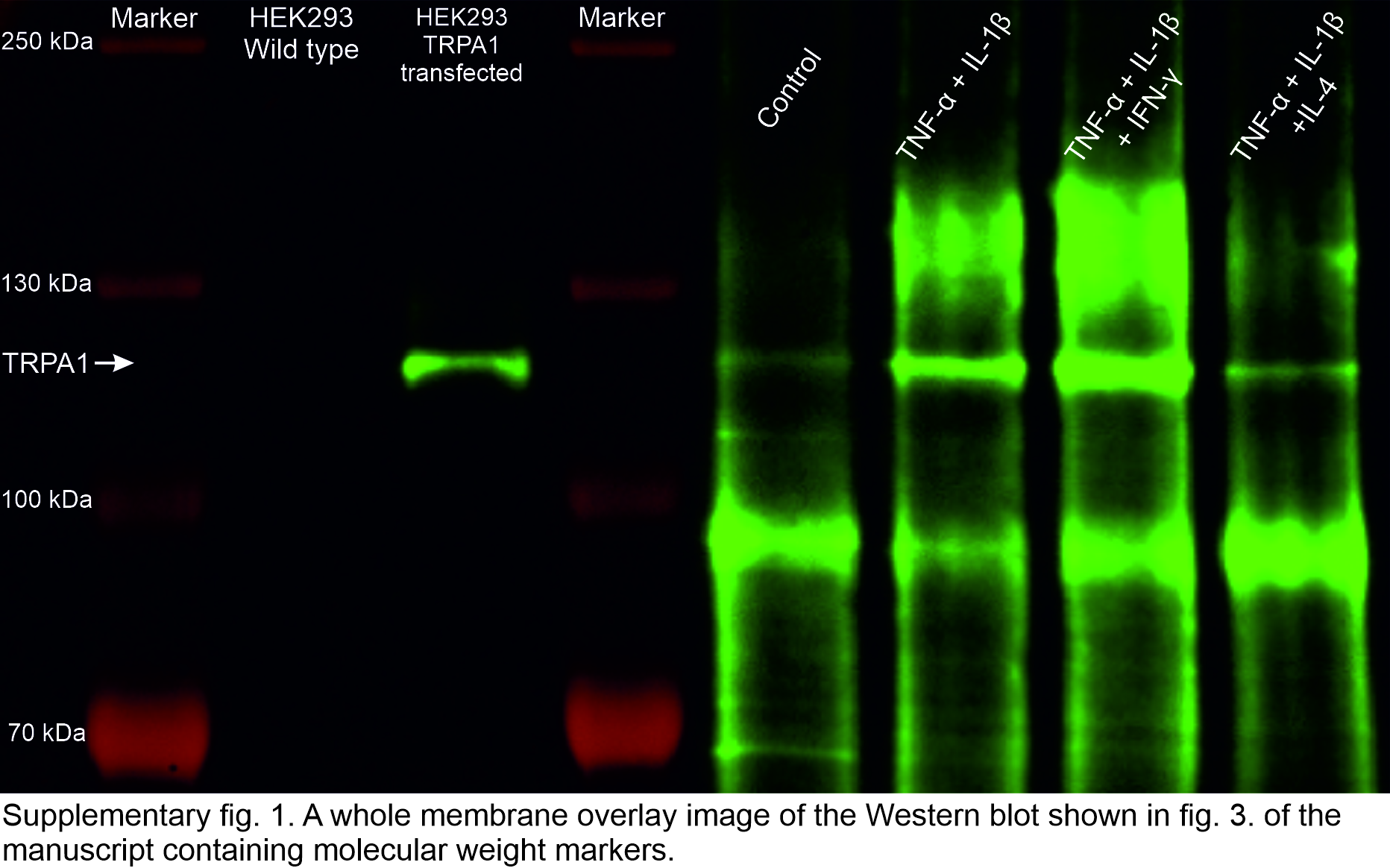

Supplement: Supplementary file 1 — Supplementary file1 (TIF 10270 KB) [file 11_2023_1750_MOESM1_ESM.tif]
